# Role of acute exposure to environmental stressors in the gut‐brain‐periphery axis in the presence of cognitive resilience

**DOI:** 10.1002/alz70860_107389

**Published:** 2025-12-23

**Authors:** Giulio Maria Pasinetti, Ruth Iban‐Arias, Nicole Less

**Affiliations:** ^1^ Icahn School of Medicine at Mount Sinai, New York, NY, USA; ^2^ James J. Peters Veterans Affairs Medical Center, Bronx, NY, USA; ^3^ James J. Peters Department of Veterans Affairs Medical Center, New York, NY, USA; ^4^ Icahn School of Medicine at Mount Sinnai, New York, NY, USA

## Abstract

**Background:**

Climate change presents an escalating threat to life and health, particularly impacting vulnerable populations. Ambient particulate matter (PM) and extreme heat stress (HS) contribute independently and synergistically to severe health problems, including respiratory and cardiovascular diseases, neurodegenerative disorders, and increased mortality rates. Particulate matter with an aerodynamic diameter of 2.5 µm or smaller (PM2.5) exacerbates conditions such as asthma, heart disease, and neurodegenerative illnesses. Meanwhile, extreme heat can lead to heat‐related illnesses, aggravate existing chronic conditions, and heighten pollution levels. The combined exposure to PM and HS intensifies these negative health effects, underscoring the urgent need for effective public health interventions and policies, particularly for the elderly population, who are at a higher risk for Alzheimer's disease.

**Method:**

In an experimental mouse model of early‐onset Alzheimer's disease (EOAD), we investigated the combined effects of exposure to extreme weather conditions, specifically ambient PM2.5 and heat stress.

**Result:**

Our findings revealed that even short‐term exposure to these environmental factors disrupts energy metabolism and mitochondrial respiratory functions, which is linked to synaptic dysfunction in the hippocampus. Notably, we identified central mechanisms associated with impaired intestinal permeability and gut dysbiosis, affecting communication along the gut‐brain axis, examined through immune and inflammatory signaling alterations. Surprisingly, despite the significant disruption of metabolic and immune‐inflammatory pathways both centrally and peripherally, we found no cognitive decline at this early stage of exposure to environmental pollutants.

**Conclusion:**

These results emphasize the urgent need to consider environmental factors in preventive public health strategies, even for individuals at risk of Alzheimer's disease who may not yet exhibit symptoms.

